# Antagonistic autoregulation speeds up a homogeneous response in *Escherichia coli*

**DOI:** 10.1038/srep36196

**Published:** 2016-10-31

**Authors:** Guillermo Rodrigo, Djordje Bajic, Ignacio Elola, Juan F. Poyatos

**Affiliations:** 1 Instituto de Biología Molecular y Cellular de Plantas, CSIC–UPV, 46022 Valencia, Spain; 2 Logic of Genomic Systems Laboratory, CNB–CSIC, 28049 Madrid, Spain

## Abstract

By integrating positive and negative feedback loops, biological systems establish intricate gene expression patterns linked to multistability, pulsing, and oscillations. This depends on the specific characteristics of each interlinked feedback, and thus one would expect additional expression programs to be found. Here, we investigate one such program associated with an antagonistic positive and negative transcriptional autoregulatory motif derived from the multiple antibiotic resistance (*mar*) system of *Escherichia coli*. We studied the dynamics of the system by combining a predictive mathematical model with high-resolution experimental measures of the response both at the population and single-cell level. We show that in this motif the weak positive autoregulation does not slow down but rather enhances response speedup in combination with a strong negative feedback loop. This balance of feedback strengths anticipates a homogeneous population phenotype, which we corroborate experimentally. Theoretical analysis also emphasized the specific molecular properties that determine the dynamics of the *mar* phenotype. More broadly, response acceleration could provide a rationale for the presence of weak positive feedbacks in other biological scenarios exhibiting these interlinked regulatory architectures.

The plastic expression of alternative phenotypes enables *Escherichia coli* to adjust to many environmental circumstances^1^. When these circumstances get particularly adverse for survival, the changes in phenotype normally involve a number of physiological responses that help the bacterium to defend against the effects of the stress^2^. Timing and variability of the response become thus essential properties to regulate.

One of these physiological programs corresponds to the multiple antibiotic resistance (*mar*) phenotype, which capacitates bacteria to tolerate several toxins including antibiotics like tetracycline or chloramphenicol^3^. That this response connected for the first time antibiotic resistance to the bacterial chromosome, rather than being caused by a plasmid-borne gene, prompted the search for a better understanding of its genetic architecture. In this way, we currently recognize that the *mar* phenotype is coupled to a unique operon architecture harboring a repressor (MarR) and an activator (MarA), and that it is additionally modulated by other transcriptional factors (e.g., SoxS or Rob)^4^,^5^,^6^. Early experiments also identified salicylates (and other repellents) as potential inducers of the phenotype^5^,^7^. The expression of the operon is then the result of the inactivation of the repressor -MarR represents the sensor of the stress- and the later boost in activator levels -MarA works as the actuator of the system. Increase of MarA abundance acts subsequently on a relatively large regulon that includes genes contributing to efflux pumps, e.g., *acrAB-tolC*^8^,^9^, membrane permeability systems, e.g., *micF-ompF*^10^, etc. Beyond all molecular details, very little is known about the *dynamic* aspects of the *mar* response, and how these aspects are ultimately determined by the particular genetic circuit that governs its action.

This circuit incorporates two feedback loops (Fig. 1) involving a crucial combination of both negative and positive autoregulation (also termed as autogenous control^11^,^12^). Notably, these two types of autoregulation were shown to provide very suitable features for stress responses, such as speedup of dynamics (negative autoregulation)^12^,^13^,^14^ or diversification of cellular behavior (positive autoregulation)^15^,^16^. How both types of control act together and what is the impact of this dual regulation for the mounting of the antibiotic resistance remains, however, an open question.

To investigate this issue, we examined the *mar* response dynamics by combining mathematical modeling with quantitative measurements in *E*. *coli*. We focused on those factors influencing both response time and variability, and further isolated the consequences of the dual feedback by restricting other regulatory links (in particular, we considered a Δ*rob* mutant throughout the study). The analysis unveils how the molecular features of the activator enhances response time while limiting the eventual production of response variability. More broadly, our study provides a good example of a relatively unexplored dynamical regime within the class of coupled positive/negative feedback architectures^17^,^18^,^19^,^20^,^21^ in which the strength of the positive loop is comparatively weak, what can represent a general design principle at work in other biological contexts.

## Results and Discussion

### Dynamics and response time

We initially studied the deterministic dynamics of the *mar* response with a bottom-up mathematical model and associated nominal parameter values (Methods and Table S1, see also Fig. S1 for a parameter sensitivity analysis of the model). An important property of the response is the time to reach an induced state after sensing the external signal. To introduce a reference for this time, we imagined first that the expression of the phenotype was driven by a constitutive mode of control (also termed non-autogenous control^22^). This system would start the production of the *mar* operon at a constant rate at time *t* = 0 (i.e., when salicylate is introduced), following an exponential increase to the steady state as (1 − *e*^−*μt*^) (*μ* being the cell growth rate). The time to reach half the steady state (*t*_50_, a typical measure of speedup) is given in this case by the cell-cycle time, log(2)/*μ*^14^,^23^ (Fig. 2A, black curve). Therefore, if we consider the cell generation time experimentally measured after induction with 0.5 mM salicylate, the constitutive control would give 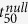 = 83.18 ± 2.09 min (null model).

The expression of the induced phenotype (0.5 mM salicylate in the following) under the dual regulation is however much faster than the null model, as predicted by the simulations (Fig. 2A in which we show explicitly the dynamics of MarR, continuous blue curve). This is also confirmed experimentally by monitoring a chromosomally integrated YFP under the control of the *mar* promoter (*P*_*mar*_: *yfp*, YFP follows the dynamics of MarR), where we obtained 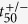 = 24.77 ± 10.04 min (+/−: dual autoregulation with positive and negative feedbacks; see also Fig. S2). The speedup could be originated by the negative autoregulation integrating the antagonistic architecture, an effect that has already been demonstrated in other contexts^13^,^14^. Indeed, by simulating the induction of a system with only the negative feedback, we predicted response speedup (Fig. 2A, dashed blue curve), what we again observed experimentally, 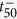 = 65.67 ± 15.66 min (−: only negative feedback, this corresponds to a Δ*marA* mutant; see also Fig. S2). However, the speedup in the latter case was significantly smaller, both according to the model predictions (Fig. 2A) and experiments (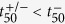, *U* − test *p* < 0.01). In consequence, and contrary to the expected delay coupled to a positive autoregulation^15^,^23^, the presence of this regulatory motif enhances the speedup of the response. Which factors determine then this feature?

### Effect of the positive feedback loop

The weak and monomeric activation of MarA^24^ are properties that generally minimize delays in expression dynamics associated to positive autoregulation. Indeed, both features lead to a linear positive feedback, with a promoter activity of 
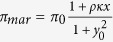
 in Eq. (4), since *κx* ≪ 1; note that here *x*, *y*_0_ represented dimensionless [MarA] and free [MarR] concentrations, respectively, while *ρ* denotes activation fold change (Methods). We thus propose that the action of MarA in the circuit represents a functional way to increase promoter strength on the fly (i.e., as long as the system is expressed). Certainly, the stronger the promoter activity, the larger the speedup due to derepression^23^. This result is captured with our simulations (Fig. 2A, in which we also show the minimal delay exhibited by a hypothetical *mar* regulatory architecture with only positive autoregulation). Moreover, the two feedbacks present as well distinct time scales: fast activation, given that MarA is actively degraded by the Lon protease^25^, and slow repression, since MarR is stable and presents a low translation rate^26^. The combination of autoregulations with distinct sign (irrespective to the stability of the proteins) originates pulses in promoter activity, which can be followed by MarA, i.e., *x* ∝ *π*_*mar*_ in a quasi-steady state situation (Fig. 2B, see also experimental data on Fig. 3A,C). According to the model, this single pulse is a consequence of an inflection point in activity, not reached when only a negative autoregulation is considered (Fig. S3).

The two time scales of the system become more explicit as we plot the response dynamics in a two-dimensional (MarA, MarR) phase space obtained with our mathematical model. Figure 3B,D show the dynamical trajectory from an uninduced steady state (no salicylate) to an induced equilibrium (salicylate either 0.5 mM or 5 mM). Note how the concentration of MarA increases fast just after induction (trajectory moves parallel to the MarA axis) to later decrease while MarR concentration grows slowly (trajectory turns left and up). This is represented in phase space by the direction and strength of the blue arrows in Fig. 3B,D. To overlay our experimental data of dynamics (Fig. 3A,C) on the phase space (blue circles in Fig. 3B,D), we obtained the MarR expression as proportional to the fluorescence levels (both are stable proteins), and the MarA expression as proportional to promoter activity (having assumed a quasi-steady state due to its short half-life; see Fig. 2B). Promoter activity was calculated by derivation of a fitted exponential equation (Methods). Theory and experiment successfully match (although our model does not fully capture the exact uninduced equilibrium). Overall, our results show that an antagonistic autoregulation specifically constituted by a weak positive and a strong negative feedbacks is a useful strategy to reach distinctly fast responses (Fig. S4 shows an exploration of this aspect as a function of the strengths of the loops).

### Cell-to-cell variability in absence of induction

We then inspected the implications of the molecular features of the *mar* regulatory architecture on the cell-to-cell variation of the response. The distinct genetic implementation by MarA and MarR of an interlinked positive and negative feedback motif presents suitable properties to reach excitable behavior (even to produce sustained oscillations) in the absence of any external signal^27^,^28^. Oscillations appear when a clear separation of time scales between activator and repressor dynamics exists. The separation implies two conditions: (i) the activator degradation should be stronger than the repressor one (in our case, MarA is quickly degraded by a protease^25^, whereas MarR is diluted due to cell growth rate), and (ii) the activator translation rate should be stronger than the repressor one (here, the RBS of MarA is stronger than the one of MarR, Supplemental information). Despite the two time scales anticipated, the monomer action of the activator (MarA) prevents however this complex behavior. This is confirmed by stochastic simulations of the model (Fig. 4A, left; see phase-space dynamics in Fig. S5A).

We also examined theoretically if the presence of alternative molecular features could cause different dynamics. For that, we introduced several modifications to the original mathematical model that either included the activator as a multimer or incorporated asymmetric and strong competitive binding (of the activator on the repressor). Both circuit variants exhibited more complex dynamics (Fig. 4A, right; see phase-space dynamics in Fig. S6). In particular, stochastic pulses can appear when MarA is considered to prevent MarR binding or when MarR is also considered short-lived^29^, even with a monomeric activator. However, previous experiments support relatively independent action of MarA and MarR^30^, aside from the fact that their operator sequences do not overlap (see Methods for further discusssion).

Experimentally, we inspected the single-cell behavior of the circuit by following YFP expression of growing bacterial microcolonies in the fluorescence microscope. Figure 4B presents a typical expression trajectory along a branch of a cell lineage tree at successive times. This indicates a noisy dynamics around a steady state (Fig. S5A,B). Moreover, much of the experimentally observed cell-to-cell variability in YFP is correlated with that of a CFP signal under a control promoter (left inset of Fig. 4B; this includes data of several lineages). The histogram of variability on YFP suggests a constant production of the proteins of the *mar* operon, and the absence of any oscillatory or pulsing dynamics (right inset of Fig. 4B and comparison with model in Fig. S5C). These results corroborate overall that it is indeed the weak strength of the positive autoregulation what determines the lack of heterogeneity.

### Cell-to-cell variability of the induction dynamics

We asked next to what extent the response upon induction with salicylate is expressed differentially, e.g., only triggered in a subset of the population^31^. This behavior is again hypothesized due to the presence of the positive feedback loop, an architecture that generally relates to heterogeneous responses even when bistability is not observed^32^. By following the dynamics of a growing population of cell lineages upon induction with salicylate (Fig. 5A), we observed that activation is relatively homogeneous, with unimodal distributions being clearly identified at different times (Fig. 5B). Because our model predicted higher variability by considering a strong activator, we attribute the observed coherent behavior across the population to the weak positive feedback (Fig. S5C,D). This is in tune with previous work showing that the presence of coupled positive and negative feedback loops contributes to reduce noise in gene expression^16^. Ultimately, our results show how a circuit integrating a strong negative feedback with a weak positive one generates a graded and relatively homogeneous response.

To quantify nevertheless the variability that we did observe in the single-cell response, we fitted the dynamics of YFP expression of each cell lineage (microcolony) to a generalized exponential model (see Methods; Fig. S7A). This fit allowed us to characterize the response time of the circuit (*t*_50_) for each lineage. We found that the resulting distribution is skewed, centered in about 2 h (Fig. 5A). Model simulations also revealed variability in the dynamic response from lineage to lineage when expressing the *mar* operon upon a common dosage (Fig. S7B), which may suggest the transient emergence of subpopulations with different antibiotic resistance levels. At the single cell level, where cells grew in solid phase and at a lower temperature, and for 5 mM salicylate, we identified a lower growth rate (giving a cell-cycle time of 2.3 h) with respect to the population level (of 1.6 h). We also identified a significant delay in the expression of the system (estimated in 0.8 h), which may reflect the time that needs salicylate to reach the cells on the agarose pads. A model simulation in the deterministic regime, rescaling the transcription rate of the system (*π*_0_) 30% according to ref. 33 and considering such a delay, recovered the mean of YFP/CFP expression (Fig. 5B). Our results finally highlight an induction dynamics faster than the one derived from the null model.

## Conclusions

**Figure 1:**
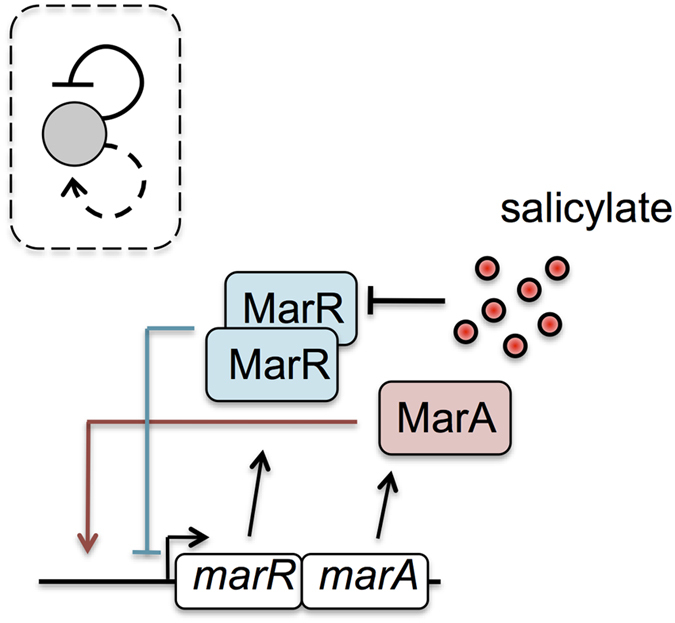
The *mar* circuit regulates the response of *Escherichia coli* to a number of toxic compounds, including antibiotics. It consists of a dual autogenous control motif constituted by an activator (MarA; acting as a monomer), and a repressor (MarR; acting as a dimer that can be inactivated by several compounds, e.g., salicylate). In more general terms (inset), the motif represents a combination of a strong (continuous line) negative and weak (dashed line) positive feedback loops. This motif is unique in the transcriptional regulatory network of *E*. *coli*.

**Figure 2:**
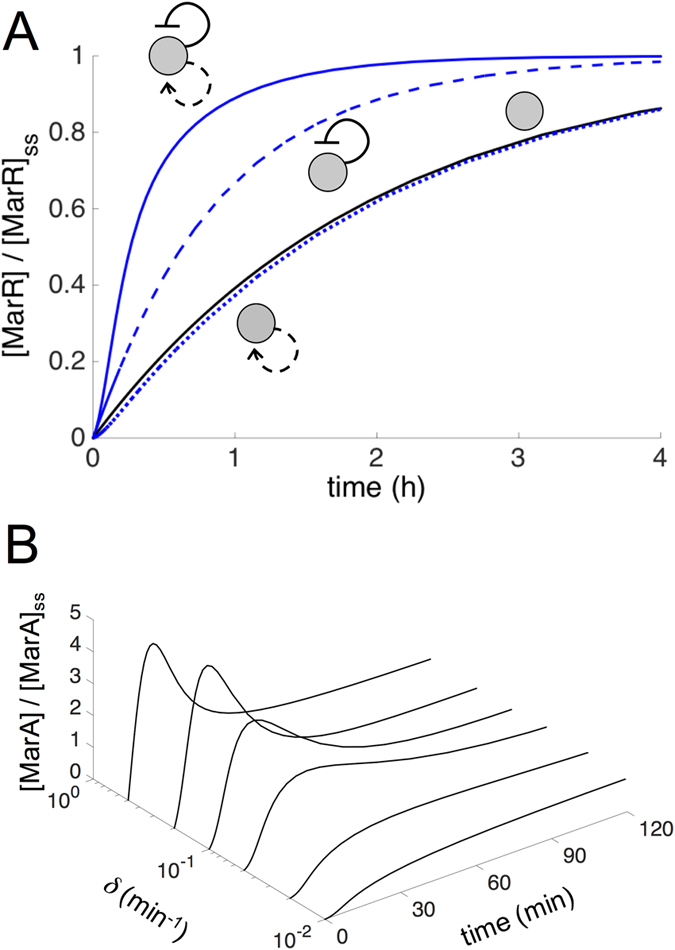
Theoretical analysis of the dynamics of the *mar* circuit and associated modifications. (**A**) MarR response upon induction with 0.5 mM salicylate normalized by the steady state (ss) value (starting from [MarR] = [MarA] = 0 in the absence of the signal). We compared the dynamics of the wild-type circuit (solid blue line) with those of two circuit variants without the positive (dashed blue line; a circuit lacking MarA) or negative (dotted blue line; a circuit lacking MarR in presence of salicylate) feedback, respectively, and with a third system exhibiting a constitutively controlled response (black line, this represents a null reference model of the dynamics, see main text). All simulations performed by using nominal parameter values (Table S1). (**B**) MarA dynamics (relative to the ss value) for different degradation rates of this protein (*δ*) upon induction with 0.5 mM salicylate. When MarA is unstable (as it is the case in the natural system), the protein dynamics *follows* the pulse-like behavior of the promoter activity (see also Fig. 3; note that the cell growth rate is *μ* < 10^−2^ min^−1^).

**Figure 3:**
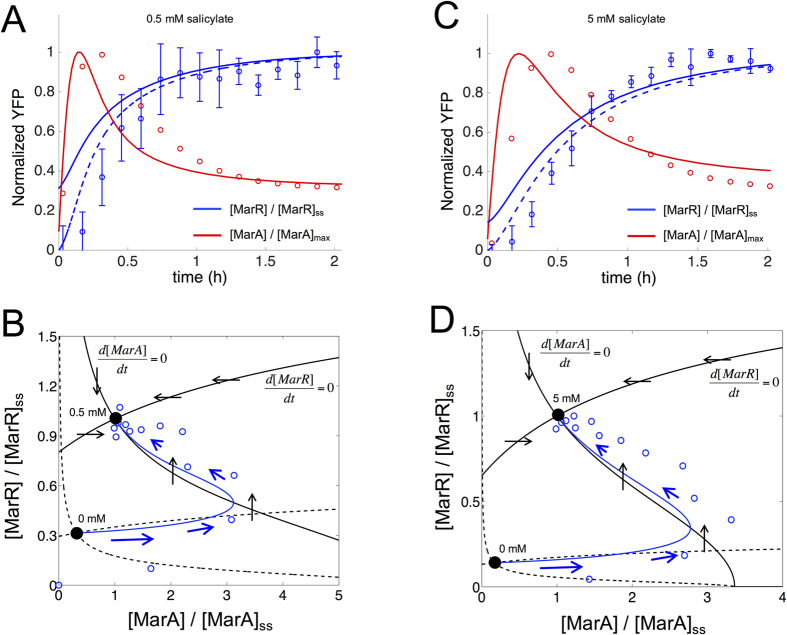
Experimental and theoretical dynamics of the *mar* circuit. (**A**,**C**) Normalized YFP expression upon induction with salicylate [0.5 mM in (**A**), and 5 mM in (**C**)] at the population level (blue circles; error bars correspond to the mean and standard deviations of three independent replicates; fluorescence values normalized by the maximum, which is the steady state value). Red circles correspond to promoter activity (normalized by the maximum) calculated from the generalized exponential model inferred from the experimental data (blue circles). Solid blue/red lines correspond to a model simulation of the *mar* circuit (blue for MarR, red for MarA) starting from the uninduced steady state. Dashed blue line correspond to a model simulation starting from [MarR] = [MarA] = 0. (**B**,**D**) Two-dimensional phase space associated to (MarA, MarR) dynamics (ss refers to steady state). Nullclines (black curves; solid for induced and dashed for uninduced situations) represent the trajectories in this space where only the concentration of MarR (*d*[MarA]/*dt* = 0 nullcline) or MarA (*d*[MarR]/*dt* = 0 nullcline) changes. Steady states (black points) are given by the intersection of these curves. A trajectory upon induction [0.5 mM in (**B**), and 5 mM in (**D**)] from the uninduced state (0 mM) is represented; blue solid line is the simulation and blue circles the experimental data. Blue arrows represent direction and strength of change (the bigger the arrow the faster the change). Black arrows represent direction of change in the nullclines of the induced situation.

**Figure 4:**
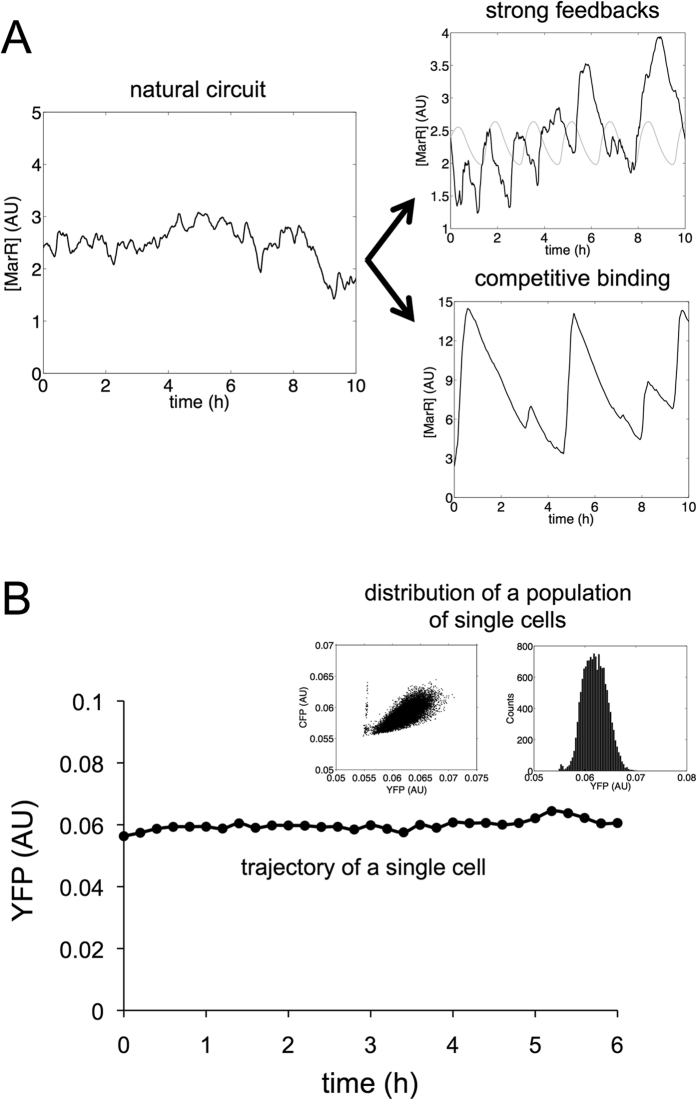
Single-cell dynamics of the *mar* circuit without being induced. (**A**) Model simulation of the stochastic dynamics of MarR [arbitrary units (AU)] in absence of salicylate (left), and also simulations when a cooperative MarA is considered (right, top; the gray curve shows the deterministic dynamics) and when MarA prevents MarR binding (right, bottom). (**B**) Representative trajectory (YFP) of a single cell. In the inset, the distribution of YFP for all single cells at all time points confirms a unimodal distribution corresponding to a continuous production of MarR [arbitrary units (AU)], and the correlation between YFP and CFP.

**Figure 5:**
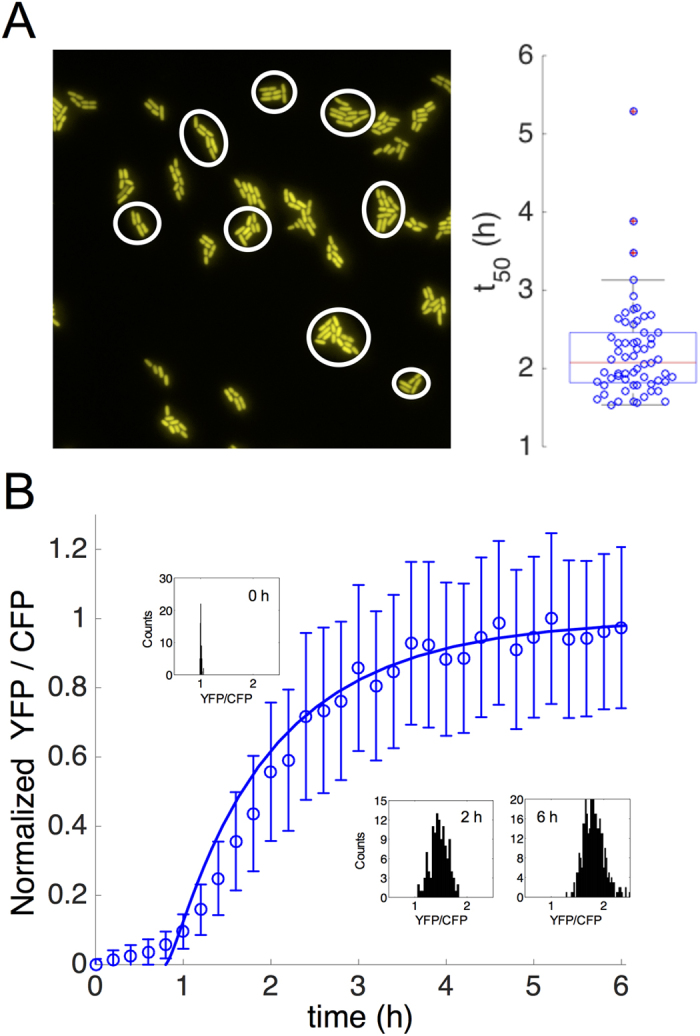
Single-cell dynamics of the *mar* response upon induction. (**A**) The image shows a subset of lineages with different YFP expression levels upon induction with 5 mM salicylate. On the right, variation in response time (*t*_50_) is quantified among 60 different lineages within the population. (**B**) Graded transcriptional activation of the *mar* phenotype, measured as the ratio YFP/CFP, in a growing population of single cells (histograms at different times illustrate the relatively homogeneous response). Solid line is the model simulation, considering a delay in expression.

**Figure 6:**
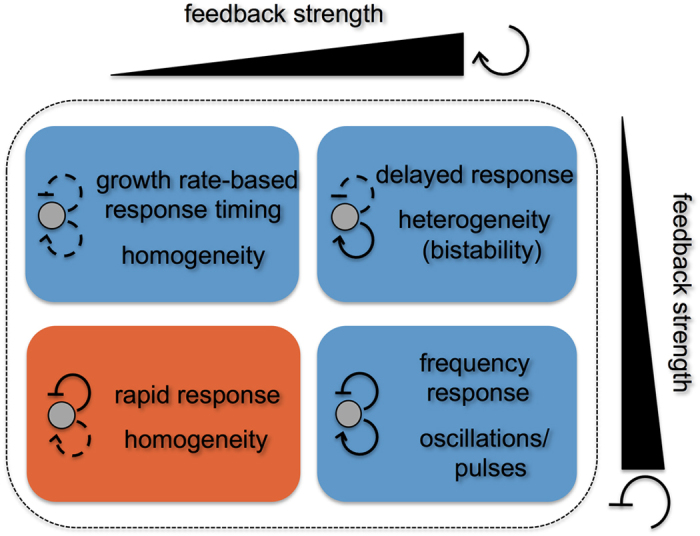
Interlinked feedback loop architectures can be generally classified according to the strength of their constituent feedbacks (represented by the ramp; dashed/continuous feedback loops correspond to weak/strong regulations, respectively). Circuits that include a strong positive feedback can originate bistable, pulsing and oscillatory gene expression programs depending on the relative strength of their constituent negative feedback. The functional role of circuits constituted by weak positive and strong negative feedbacks (orange domain) is less known (study of this work). The dual autoregulation of the *mar* phenotype corresponds to this regime, which exhibits a homogeneous but rapid response.

The *mar* stress-response system presents a unique antagonistic autoregulatory architecture within *E*. *coli*’s transcriptional network^34^. We showed that in this architecture, the presence of a weak positive feedback (through MarA) (Fig. 1) serves to increase the transcription rate on the fly due to the derepression of the system (through MarR) and the concomitant expression of the activator. This impacts the response by accelerating its dynamics and by limiting the heterogeneity of the induced phenotype within a population (Figs 3 and 5), a behavior that is true for independent or symmetric competitive binding of the regulators (Fig. 2A and Fig. S8). Coordination between repressor and activator generates pulses in promoter activity, which can be followed by an actuator with a short half-life (like MarA). The expected delay associated to positive feedbacks is thus minimized in this domain of weak activation and monomeric action of the regulator. These attributes additionally limit large-amplitude (and heterogeneous) transient responses, and ultimately set apart the observed dynamics from other potential behaviors (Fig. 4). Thus, we can present this system as a prototypical example of a subclass of regulatory topologies -within those combining positive and negative feedbacks- in which the positive loop is comparatively weak (Fig. 6). In contrast to the delays and heterogeneity typically observed with strong positive feedbacks (see also Fig. S9), this subclass gives rise to a particularly strong acceleration of the response that is expected to be observed in similar genetic control architectures of other organisms.

## Methods

### Strains, culture media and reagents

Two-color fluorescent reporter *E*. *coli* strains (IE01, IE02, TC01 and TC02) were engineered to measure the activity of the *mar* promoter. The strain IE01 contains a chromosomal copy of the *yfp* gene under the control of the *mar* promoter, and the *cfp* gene expressed with a constitutive promoter. The strains IE02 and TC01 were constructed by deletion on IE01 of the *rob* and *marA* genes, respectively, with the application of a standard knockout protocol^35^. The strain TC02 was constructed as the double knockout of *rob* and *marA* genes. Note that we only considered strains IE02 (Δ*rob*) and TC02 (Δ*rob*Δ*marA*) to characterize the dynamics of the antagonistic autoregulatory motif. Medium LB was always used for overnight cultures. Minimal medium M9 (M9 salts 1x, MgS_4_ 2 mM, CaCl_2_ 0.1 mM, glucose 0.4%, casamino acids 0.05%, vitamine B1 0.05%) was used to grow cells during characterization experiments. Glucose inhibits the production of internal cAMP, so the circuit is decoupled to the CRP regulation^36^. To induce the *mar* circuit, different concentrations of salicylate (Sigma Aldrich) were used. When appropriate, kanamycin was used at 50 *μ*g/mL. See Supplementary Information for extended experimental procedures.

### Quantification of fluorescence in a cell population

Cultures (2 mL) inoculated from single colonies (three replicates) were grown overnight in LB medium supplemented with glucose 0.4% at 37 °C and 170 rpm. The cultures were next diluted 1:200 in M9 minimal medium and grown for 2 h at the same conditions to then load the wells (200 *μ*L) of the microplate (Thermo Scientific) with salicylate when appropriate. The microplate was assayed in a Victor X2 (Perkin Elmer) measuring absorbance (600 nm), YFP (497/16 nm, 535/40 nm), and CFP (434/17 nm, 479/40 nm) for 3 h at 37 °C with shaking (Fig. S10). During this period, cell growth remained in exponential phase (Fig. S11; *μ* ≃ 0.5 h^−1^). Analysis of fluorescence data is further described in Supplementary Information.

### Quantification of fluorescence in single cells

A culture (2 mL) inoculated from a single colony was grown overnight in LB medium supplemented with glucose 0.4% at 37 °C and 170 rpm. The culture was then diluted 1:200 in M9 minimal medium and grown for 4 h at the same conditions. A 1:10 dilution of this (2 *μ*L) was next used to load the agarose pad. Before characterization, we added 5 mM salicylate to induce cells. Agarose pads were monitored in an inverted microscope Axiovert200 (Zeiss) with objective 100X/1.45 oil Plan-Fluar at 30 °C. Cell images were acquired from the bright-field and fluorescence channels, YFP (490–510 nm, 510–560 nm) and CFP (426–446 nm, 460–500 nm), using software MetaMorph (Universal Imaging). Analysis of single cell images described in Supplementary Information.

### Bottom-up mathematical model of the mar circuit

We introduced a system of ordinary differential equations based on the known topology of the circuit (Fig. 1). MarR and MarA (together with a third protein, MarB) form an operon controlled by promoter *P*_*mar*_^37^,^38^. MarR represses *P*_*mar*_, which can be modulated by salicylate^5^, whereas MarA activates it^39^. Therefore, we could write


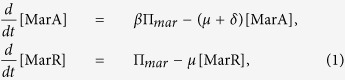


and similarly the equation for the yellow fluorescent protein (YFP) used as reporter under the control of *P*_*mar*_,


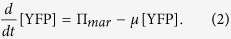


In these expressions, *μ* is the cell growth rate, *δ* the degradation rate of MarA (*δ* ≫ *μ*), noting that MarA is quickly degraded by Lon protease^25^, *β* the fold increase of MarA translation rate, noting that the ribosome binding site (RBS) is much stronger in case of the activator than the repressor and Π_*mar*_ the activity of promoter *P*_*mar*_. The equation for Π_*mar*_ can be described by means of Hill functions^40^, knowing that MarA acts as a monomer whereas MarR works as a dimer^39^, as


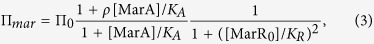


where *K*_*A*_ and *K*_*R*_ are the effective dissociation constants for transcription regulation, *ρ* the activation fold change, and Π_0_ the basal protein synthesis rate. In addition, MarR_0_ is the non-oxidized MarR by salicylate (*S*)^41^, and we can write 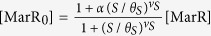, where *θ*_*S*_ is the effective dissociation constant between salicylate and MarR, *ν*_*S*_ the Hill coefficient, and *α* the minimal fraction of non-oxidized MarR.

We can further simplify the system by considering dimensionless concentrations denoted as *x* = [MarA]/*K*_*R*_, *y* = [MarR]/*K*_*R*_, and *y*_0_ = [MarR_0_]/*K*_*R*_. The previous equations now read


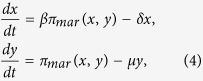


with 
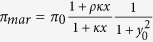
 and 
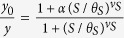
. Here *π*_0_ = Π_0_/*K*_*R*_, and *κ* = *K*_*R*_/*K*_*A*_. To compute the dynamics of a Δ*marA* system, we fixed *x* = 0, and of a system with all MarR oxidized by salicylate, we fixed *y*_0_ = 0. Nominal parameter values for this model are presented in Table S1. The preceding framework can be extended to account for the mRNA dynamics (validity of the model with only protein dynamics, Fig. S12), and the inherent stochasticity of biological systems by using a Langevin approach^42^. See Supplementary Information for details.

### Modifications of the bottom-up mathematical model

We introduced some modifications in the model to account for different molecular features. Note first that in the initial formulation of the model, Eq. (4), we assumed independent binding on the promoter of MarR and MarA. However, it may exist some competition between these two proteins^30^,^43^ what lead us to write


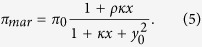


This alternative model showed similar dynamics (Fig. S8). We also considered regulation with multimers (in particular, tetramers) for both feedback loops. Thus, promoter activity was rewritten as


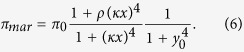


In addition, we considered a scenario of asymmetric competitive binding of MarA on MarR (i.e., the activator wins out the repressor, a condition to get pulsatile dynamics^29^). Thus, promoter activity was in this case rewritten as


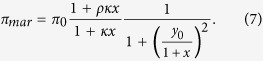


In case of the single-cell dynamics, growth rate was slower as cells grew in solid phase, and temperature was 30 °C. To apply the model we adjusted the value of *π*_0_, which controls for the transcription rate of the system. The nominal value of *π*_0_ is 0.1 min^−1^, which was estimated to fit the population dynamics. Because transcription rate increases with growth rate^33^,^44^, we wrote 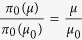. Accordingly, *π*_0_ is lower in case of single cell dynamics; in particular *π*_0_ = 0.07 min^−1^ with a ratio of growth rates of 0.7 (0.43 h^−1^ for population assays and 0.30 h^−1^ for single cell assays with 5 mM salicylate). We neglected this effect of *μ* on *π*_0_ in case of population dynamics for different salicylate dosages.

### Empirical fitting of the dynamic behavior

We applied a generalized exponential model,


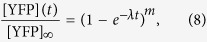


to fit the experimental response of the system upon induction with salicylate, at both population and single cell levels (*λ* and *m* are two parameters that need to be fitted in each case). With this model, one can compute the response time (time to reach half of the steady state value of expression) as 
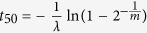
. Bootstrapping was applied to calculate the errors associated to the measurements of response time, and *U*-tests were performed to compare distributions of inferred parameters. Moreover, promoter activity was calculated empirically by derivation of the equation for [YFP](*t*)^45^, Eq. (8).


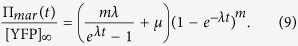


## Additional Information

**How to cite this article**: Rodrigo, G. *et al*. Antagonistic autoregulation speeds up a homogeneous response in *Escherichia coli*. *Sci. Rep*. **6**, 36196; doi: 10.1038/srep36196 (2016).

**Publisher’s note**: Springer Nature remains neutral with regard to jurisdictional claims in published maps and institutional affiliations.

## Supplementary Material

Supplementary Information
