# Supplementary material for: Antagonistic autoregulation speeds up a homogeneous response in Escherichia coli
Source: Sci Rep. 2016 Oct 31;6:36196. doi: 10.1038/srep36196 (PMC5086920; doi:10.1038/srep36196)
Supplement: Supplementary Information [file srep36196-s1.pdf]

Supplemental Information

Antagonistic autoregulation speeds up a homogeneous response in *Escherichia coli*

Guillermo Rodrigo, Djordje Bajić, Ignacio Elola, and Juan F. Poyatos

Table of Contents

Supplementary Experimental Methods……………………………………….…..2
Strains construction
Culture media and reagents
Quantification of fluorescence in a cell population
Analysis of fluorescence data
Quantification of fluorescence in single cells
Analysis of single cell images

Supplementary Mathematical Methods……………………………………………...8
Bottom-up mathematical model
Simplification of the mathematical model
Stochastic modeling

Supplementary Figures and Figure Legends………………………………………..12

Supplementary Tables……………………………………………………………27

Supplementary References………………………………………………………28

Supplementary Experimental Methods

Strains construction

We engineered a two-color fluorescent reporter strain (*Escherichia coli*) to measure the activity of the *marRAB* promoter, following the work by Miyashiro & Goulian (2007)[1]. The strain (named as IE01) contains a chromosomal copy of the *yfp* gene (Yellow Fluorescent Protein) under the control of the *marRAB* promoter, and the *cfp* gene (Cyan Fluorescent Protein) expressed with a constitutive *tetA* promoter at the attachment sites in *E. coli* of lambda and HK022 phages, respectively.

To construct the YFP reporter, we amplified the promoter region of the *marRAB* operon (1616883-1617144; *E. coli* K-12 substr. MG1655; NCBI ref. NC_000913) by PCR. The primers used were: 5'-TAGCAGAATTCCGGCAGCAAC (forward), and 5'-GCATAGGGATCCTGGCAAGTAAT (reverse). The PCR product was then cloned it into a plasmid (pTM74; Miyashiro & Goulian, 2007) that contains a multicloning site upstream of the promoterless *yfp* gene to yield the plasmid pJFP01 (this work). This plasmid was integrated as single copy into the lambda phage attachment site of *E. coli* MG1655 using the helper plasmid pInt-TS (Weiss *et al.*, 1999). The residual *cat* gene linked to the *marRAB* promoter was replaced by *kan* by electroporating a PCR product created from the template plasmid pKD4 into the appropriate strain carrying plasmid pKD46 to yield the strain JFP01 (this work). The two-color reporter strain JFP02 was created by moving the previous marker into strain TIM64 (a strain derived from a MG1655 that constitutively expresses CFP; TIM64 does not present any antibiotic resistance marker; Miyashiro & Goulian, 2007) by P1 transduction.

[1] We thank Tim Miyashiro for strains, and Mark Goulian for strains and experimental advice.

Removal of the *kan* marker using plasmid pCP20 originated *E. coli* strain IE01. This was verified by PCR. In addition, *E. coli* strain IE02 was constructed by deletion of the *rob* gene in IE01 with the application of a Datsenko & Wanner (2000) knockout protocol followed by the removal of the *kan* marker using plasmid pCP20 (Datsenko & Wanner, 2000). The primers used were: 5'-ATATCCCAATGGCATCGTAAAGAACATTTTGAGGCATTTCAGTCAGTTGC GCTGGAGCTGCTTCGAA (forward), and 5'-ATGAACCTGAATCGCCAGCGGCATCAGCACCTTGTCGCCTTGCGTATAAT ATGAATATCCTCCTTAG (reverse). The sequence of the resulting strain was verified by PCR.

We also constructed two strains deleting the *marA* gene (named as TC01 and TC02)[2]. This was done from strains IE01 and IE02, respectively, using a variant of the Datsenko & Wanner (2000) knockout protocol, with the insert (*kan* marker with *marA* flanking regions) obtained by PCR from a KEIO collection strain (Baba *et al.*, 2006). Strain IE01 (or IE02) containing the pKD46 plasmid was electroporated with this insert in presence of arabinose, allowing recombination in the *marA* locus. The primers used to obtain the insert were: 5'-CGAACCCGAATGACAAGC (forward), and 5'-GCTATTGCGGATGAAAGTGG (reverse). Correct substitution of the *marA* gene by *kan* marker was checked by PCR.

Culture media and reagents

Medium LB was always used for overnight cultures. Minimal medium M9 (M9 salts 1x, $MgSO_4$ 2 mM, $CaCl_2$ 0.1 mM, glucose 0.4%, casamino acids 0.05%, vitamine B1 0.05%) was used to grow cells during characterization experiments. To induce the

[2] We thank Teresa Cordero and Jerónimo Rodríguez-Beltrán for practical assistance.

mar circuit, we used different concentrations of Salicylate (Sigma), resulting in a gradient ranging from 0 to 5 mM. When appropriate, we used kanamycin at a concentration of 50 μg/mL.

Quantification of fluorescence in a cell population

Experiments of induction were carried out to study the dynamics of the system. Cultures (volume of 2 mL) inoculated from single colonies (three replicates) were grown overnight in LB medium supplemented with glucose 0.4% at temperature of 37 ºC and shaking of 170 rpm. Cultures were then diluted 1:200 in M9 minimal medium (10 μL of culture into a final volume of 2 mL) and were grown for 2 h at temperature of 37 ºC and shaking of 170 rpm. Cultures were then used to load the multiwell plate (Thermo) with final volumes of 200 μL. Per well, we added directly a volume of the culture and a volume of salicylate stock[3].

The set up for the Victor X2 was as follows. OD_{600} measured with absorbance filter of 600 nm (0.5 s for reading). YFP measured with excitation filter of 497/16 nm, and emission filter of 535/40 nm. CFP measured with excitation filter of 434/17 nm, and emission filter of 479/40 nm. For both YFP and CFP, we took 8000 for energy lamp, and 0.1 s for counting. Temperature was at 37 ºC. The

[3] We also used mineral oil for characterization. Cultures were grown as previously indicated, and then were used to load the multiwell plate (Thermo) with final volumes of 250 μL. Per well, we added directly 200 μL of culture (counting the volume of inducers), and 50 μL of mineral oil (Sigma). However, we found that the *mar* circuit does not respond to salicylate in partially anaerobic conditions. This may be explained, at least in part, by the fact that copper appears to shift intracellularly from Cu^{2+} to Cu^{+} in absence of oxygen (Rensing & Grass, 2003), but also because fluorescent proteins require oxygen to form the chromophores (Drepper et al., 2007). Note that previous experiments in partially anaerobic conditions like ours, but with another gene circuit, have shown expression with a GFP (Setty et al., 2003).

program started first with OD_{600}, then YFP, and finally CFP, followed by 30 s of shaking in orbital mode. Then it waited for 5 min, and it started again.

Analysis of fluorescence data

We collected time-course data of fluorescence (YFP and CFP) and absorbance. Background values of absorbance and fluorescence, which corresponded to M9 minimal medium, were subtracted to correct the signals. The normalized fluorescence (for both YFP and CFP) was calculated as the ratio of fluorescence and absorbance. Similar values of normalized fluorescence were reported for MG1655 cells and for M9 minimal medium, which indicated that the auto-fluorescence of cells was negligible in this case. The growth rate of cells was calculated as the slope of the linear regression between the log of corrected absorbance and time in exponential phase. Time-dependent promoter activity, defined as the instantaneous production rate of normalized YFP fluorescence (magnitude per cell), was calculated for each time point using the derivative of the normalized fluorescence. Promoter activity in steady state, defined as the stationary production rate of normalized YFP fluorescence (magnitude per cell) in exponential phase, was calculated as the average over time (for $t > 2$ h) of normalized fluorescence times growth rate. The error associated to that measure was obtained by calculating the standard deviation over replicates in all time points, then squaring all these deviations to average them, and finally getting the root square. Data were analyzed with Matlab (MathWorks).

Quantification of fluorescence in single cells

Experiments of induction were carried out to study the heterogeneity of the dynamic response. Culture (volume of 2 mL) inoculated from a single colony was grown

overnight in LB medium supplemented with glucose 0.4% at temperature of 37 ºC and shaking of 170 rpm. Culture was then diluted 1:200 in M9 minimal medium (10 μL of culture into a final volume of 2 mL) and was grown for 4 h at temperature of 37 ºC and shaking of 170 rpm. The different cultures diluted 1:10 were then used to load the agarose pads.

Agarose pads were prepared with a volume of 5 mL of M9 minimal medium and 0.075 g of agarose. It was dissolved by vortexing and microwaving. The pads were then allowed to solidify for about 1 h at room temperature before seeding bacteria. 2 μL of each culture were then used to load the pads. They were kept for about 15 min at room temperature so that cells can be absorbed into the agarose. Just before characterization with the microscope, 2.5 μL salicylate from a solution 0.1 M was used to induce cells in solid medium, having estimated the volume of the agarose pad in 50 μL (resulting concentration of salicylate about 5 mM).

In each pad, fields with an adequate initial density of cells were chosen, the first photo was taken, and salicylate was added. Photos were taken for each field every 12 minutes.

Agarose pads were monitored in an inverted microscope Axiovert200 (Zeiss) with objective 100X/1.45 oil Plan-Fluar at temperature of 30 ºC. The microscope was equipped with a digital camera C9100-02 (Hamamatsu), a Xenon lamp XBO 75W/2, an optical filter changer Lambda 10-2 (Sutter), a motorized stage (Marzhauser), and a temperature controller (Cell Observer-Zeiss). Moreover, the microscope was automated by the commercial software MetaMorph (Universal Imaging). Cell images were acquired from the bright-field and fluorescence channels. We used the fluorescence filters yellow FP (490-510 nm, 510-560 nm) and cyan FP (426-446 nm, 460-500 nm).

Analysis of single cell images

Microscopy photos were segmented and analyzed using the EBImage package for R from Bioconductor. Segmentation allowed us to identify sets of pixels belonging to individual cells, and measure the average apparent YFP and CFP intensities for each of these sets. YFP or the ratio YFP/CFP was used as proxy for system activity. It was normalized by subtracting the initial value, and then dividing by the final value, obtaining a dynamics that goes from 0 to 1.

Supplementary Mathematical Methods

Bottom-up mathematical model

We constructed a system of ordinary differential equations (ODEs) by knowing the topology of the circuit. MarR, MarA and MarB form an operon controlled by promoter P_{mar} (Alekshun & Levy, 1997; Chubiz *et al.*, 2012). MarR represses P_{mar}, which can be modulated by salicylate (Cohen *et al.*, 1993). MarB also represses P_{mar} (Vinué *et al.*, 2013), whereas MarA activates it (Martin *et al.*, 1996). On the other hand, Rob is controlled by promoter P_{rob}, and it activates P_{mar} (Alekshun & Levy, 1997). MarA and Rob repress P_{rob} (Schneiders & Levy, 2006). Finally, in our system YFP models a downstream gene controlled by promoter P_{mar}. Although MarR, MarA and MarB are transcribed from the same promoter, the corresponding protein expressions may be different each other due to distinct translation rates. By analyzing the 5' untranslated regions of MarR, MarA and MarB with RBS calculator (Salis *et al.*, 2009), considering the 30 nucleotides upstream and the 7 nucleotides downstream of the start codon, we found that translation rates of MarA and MarB are about 30-fold and 20-fold, respectively, higher than the translation rate of MarR. This is in tune with previous experimental observations (Martin & Rosner, 2004). In addition, promoter P_{mar} is regulated by CRP-cAMP. Here, we do not consider the moderate activation of Rob by salicylate (Chubiz *et al.*, 2012), because this protein is highly expressed and then a moderate increase in it will not significantly impact the results. Therefore, we could write

$$\frac{d}{dt}[MarA] = \beta\Pi_{mar} - (\mu + \delta)[MarA]$$
$$\frac{d}{dt}[MarR] = \Pi_{mar} - \mu[MarR]$$
$$\frac{d}{dt}[MarB] = \beta^{*}\Pi_{mar} - \mu[MarB] \qquad \text{(S1)}$$
$$\frac{d}{dt}[Rob] = \Pi_{rob} - \mu[Rob]$$
$$\frac{d}{dt}[YFP] = \Pi_{mar} - \mu[YFP],$$

where μ is the cell growth rate, δ the degradation rate of MarA ($\delta >> \mu$), noting that MarA is quickly degraded by protease Lon (Griffith *et al.*, 2004), β (and β^{*}) the fold increase of MarA (MarB) translation rate, and Π_{mar} and Π_{rob} the activity of promoters P_{mar} and P_{rob}, respectively.

In this work, we considered a Δ*rob* scenario to precisely study the antagonistic autoregulatory motif implemented by MarR and MarA. Also, we neglected the effect of MarB[4]. The equation for Π_{mar}, knowing that MarA acts as a monomer whereas MarR as a dimer (Martin *et al.*, 1996), could be approached by a Hill function (Bintu *et al.*, 2005). Therefore, it turned out

$$\Pi_{mar} = \Pi_0 \frac{1 + \rho[MarA]/K_A}{1 + [MarA]/K_A} \frac{1}{1 + \left([MarR_{free}]/K_R\right)^2}, \qquad \text{(S2)}$$

where K_A, and K_R are the effective dissociation constants for transcription regulation, and ρ the activation fold change. Π_0 is the basal protein synthesis rate.

In addition, we have

$$[MarR_{free}] = \frac{1 + \alpha\left([Sal]/\theta_S\right)^{v_S}}{1 + \left([Sal]/\theta_S\right)^{v_S}}[MarR], \qquad \text{(S3)}$$

where θ_S is the effective dissociation constant between salicylate (Sal) and MaR, v_S the Hill coefficient, and α the minimal fraction of free MarR.

---

[4] The effect of MarB is weak, and can then be assumed dispensable in presence of MarR. In our model, even for high levels of salicylate, there is a remnant amount of free MarR, parameterized by α.

Simplification of the mathematical model

Our bottom-up mathematical model can be simplified for a better analysis of the dynamic response. By noting $x = [MarA]/K_R$, $y = [MarR]/K_R$, $y_0 = [MarR_{free}]/K_R$ (the concentration of MarR is equal to the one of YFP), $\pi_0 = \Pi_0/K_R$, and also $\kappa = K_R/K_A$, we could write a simplified system of ODEs. Thus, we obtained

$$\begin{aligned} \frac{dx}{dt} &= \beta\pi_{mar} - \delta x \\ \frac{dy}{dt} &= \pi_{mar} - \mu y \\ \pi_{mar} &= \pi_0 \frac{1+\rho\kappa x}{1+\kappa x} \cdot \frac{1}{1+y_0^2}, \end{aligned} \qquad \text{(S4)}$$

where MarA could be approached to a quasi-steady state ($x \propto \pi_{mar}$, a function of time). And also we had

$$\frac{y_0}{y} = \frac{1+\alpha\left([Sal]/\theta_S\right)^{\nu_S}}{1+\left([Sal]/\theta_S\right)^{\nu_S}}. \qquad \text{(S5)}$$

In case of maximal induction of the system with salicylate, α modulates the regulatory role of MarR. To obtain dimensional parameters, see Table S1 for values of K_R and K_A.

An extended model including mRNA

Our bottom-up mathematical model can be extended to account for the mRNA (r), the same molecule for MarA and MarR. Thus, we wrote

$$\begin{aligned} dr/dt &= 4\delta(\pi_{mar} - r) \\ dx/dt &= \beta r - \delta x \\ dy/dt &= r - \mu y \end{aligned} \qquad \text{(S6)}$$

having assumed a degradation rate of the mRNA of 4δ. Note that if we consider $dr\,/\,dt = 0$, we recover the previous model.

Stochastic modeling

Using a Langevin approach[5], our ODE-based mathematical model can be extended to account for the inherent stochasticity of biological systems. Because in bacteria noise has two components (intrinsic and extrinsic) and is predominantly generated at the transcription level (Swain *et al.*, 2002), we considered a stochastic process ξ_s (where s is the inverse of the correlation time) with statistics $\langle \xi_s(t) \rangle = 0$ and $\langle \xi_s(t')\xi_s(t'+t) \rangle = \frac{s}{2}e^{-s|t|}$. With $s = \delta$, we have the stochastic process that describes intrinsic noise (very rapid fluctuations), while with $s = \mu$, we have the one that describes extrinsic noise (slow fluctuations). Thus, and having assumed similar mRNA degradation and MarR translation rates [note that ξ_δ is a stochastic process associated to intrinsic noise at the mRNA level, which is translated to generate intrinsic noise at the protein level; see Rodrigo *et al.* (2013)], we can write

$$\begin{aligned}\frac{dx}{dt} &= \beta\pi_{mar} - \delta x + \beta\sqrt{\frac{2\pi_{mar}}{K_R}}\xi_\delta(t) + \beta q \xi_\mu(t) \\ \frac{dy}{dt} &= \pi_{mar} - \mu y + \sqrt{\frac{2\pi_{mar}}{K_R}}\xi_\delta(t) + q\xi_\mu(t)\end{aligned}, \qquad \text{(S7)}$$

where q is the extrinsic noise magnitude. This system can be solved numerically by following the method described by Rodrigo *et al.* (2011).

[5] The Langevin approach is based on the construction of an Itô stochastic differential equation (Gillespie, 2000). The master equation that is useful to represent the stochasticity of biochemical systems is indeed equivalent to the Langevin equation for an appropriate random force (Bedeaux, 1977). The Langevin approach, however, allows maintaining the ODE formalism and even introducing easily noises with particular correlation times.

Supplementary Figures and Figure Legends

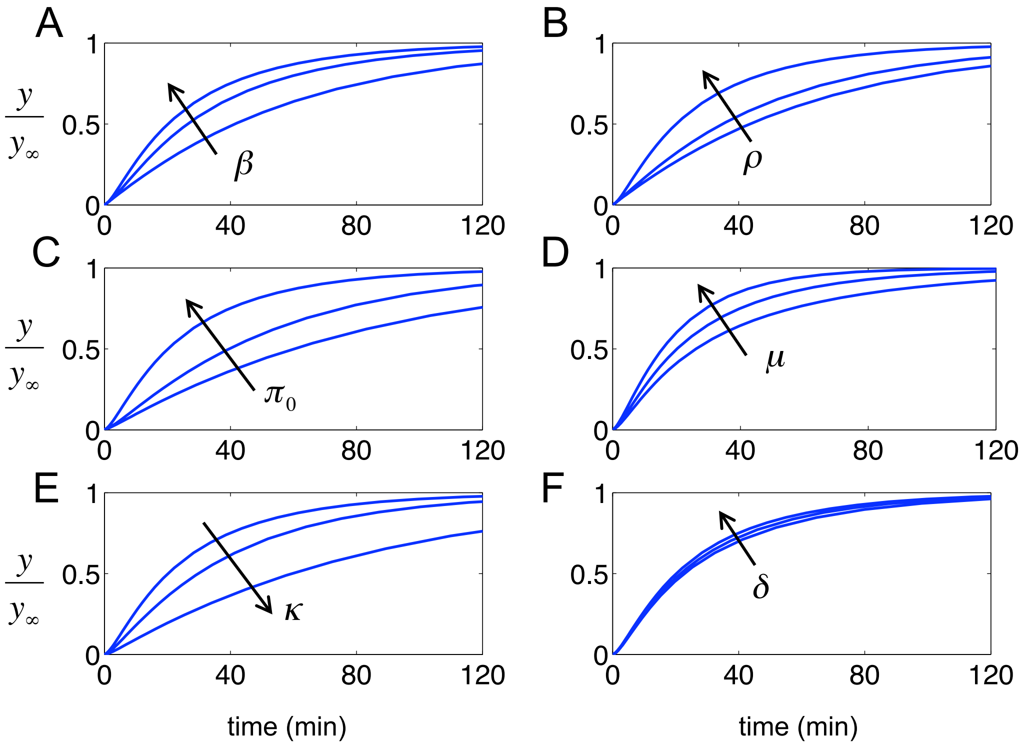

Figure S1: Parameter sensitivity analysis of our mathematical model of antagonistic autoregulation.

(A - F) Computational simulations of the dynamic behavior with toy parameter values. Model-based sensitivity analysis of various parameters. (A) β = 30 / 10 / 1. (B) ρ = 5 / 2 / 1. (C) π_0 = 1 / 0.4 / 0.2 min^{-1}. (D) μ = 0.02 / 0.01 / 0.005 min^{-1}. (E) κ = 0.01 / 0.02 / 0.1. (F) δ = 0.5 / 0.1 / 0.01 min^{-1}. For parameters not specified, we took the following parameter values: δ = 0.5 min^{-1}, μ = 0.01 min^{-1}, π_0 = 1 min^{-1}, ρ = 5, κ = 0.01, β = 30, θ_S = 0.1 mM, ν_S = 2, and α = 0.01.

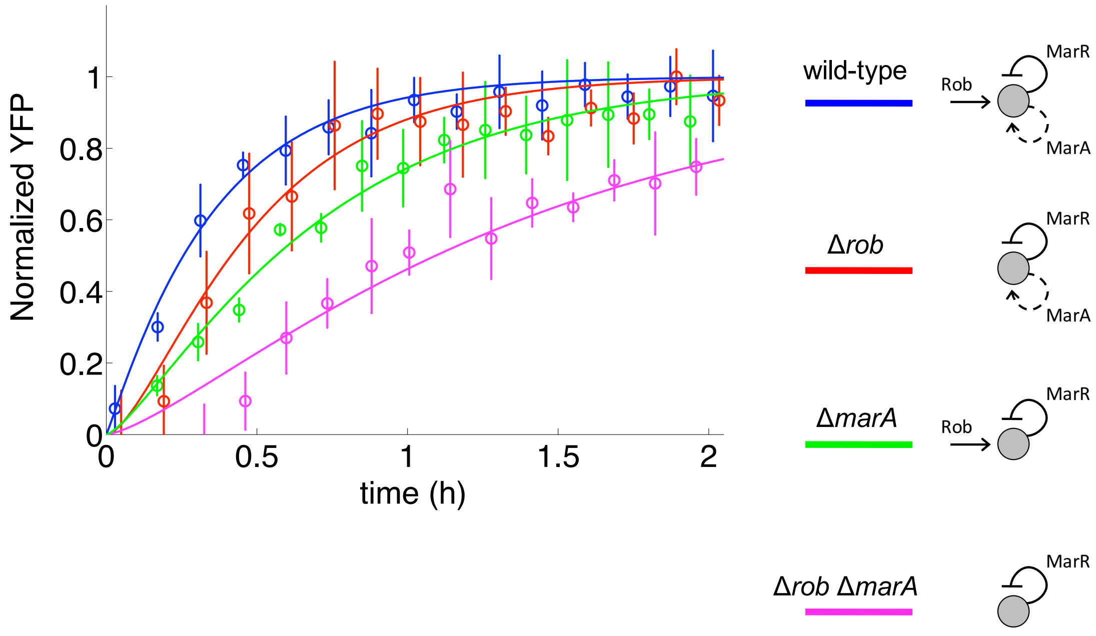

Figure S2: Dynamics of different feedback-based systems based on the *mar* circuit.

Dynamic response upon induction with 0.5 mM salicylate (error bars correspond to the mean and standard deviations of three independent replicates; fluorescence values normalized by the maximum). Continuous lines are fittings to $\left(1-e^{-\lambda t}\right)^{m}$. We obtained the following response times: t_{50} = 15.35 min for the wild-type system (IE01 strain), t_{50} = 24.77 min for the Δ*rob* system (IE02 strain), t_{50} = 33.79 min for the Δ*marA* system (TC01 strain), and t_{50} = 65.67 min for the Δ*rob*Δ*marA* system (TC02 strain). In case of the TC02 strain, our fluorometer had not sufficient precision to report fluorescence values above the background at initial times, because the two activators of the promoter (MarA and Rob) here do not exist and then the expression levels are lower. This may lead to take with caution the inferred value of t_{50} for the Δ*rob*Δ*marA* system. Anyway, model simulations indicate that t_{50} correlates negatively with promoter activity (i.e., stronger the promoter, faster the response). Promoter activity is highest in the wild-type system, then higher in the Δ*rob* system

than in the Δ*marA* system, and finally lowest in the Δ*rob*Δ*marA* system; in tune with the inferred values of t_{50}.

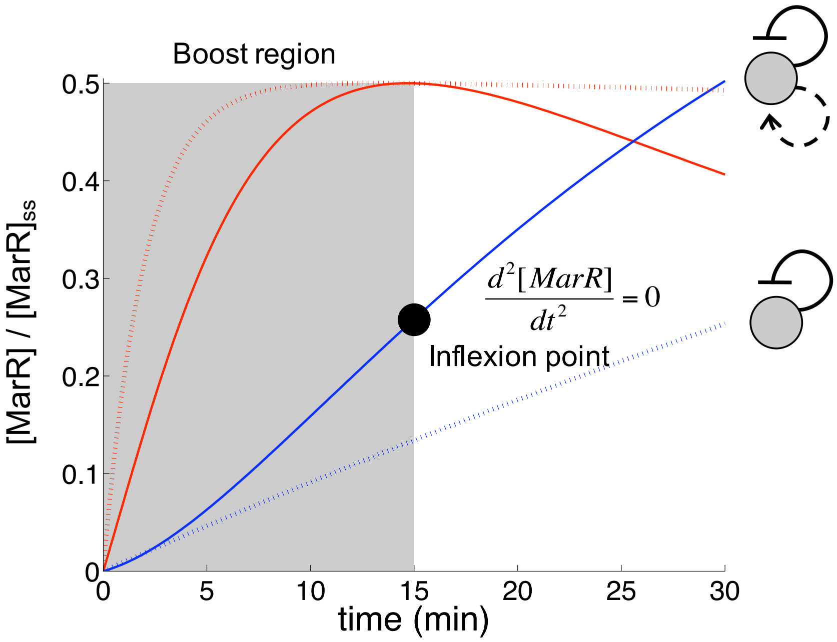

Figure S3: Effect of positive autoregulation within the antagonistic control of the *mar* response.

Controlled comparison between the dynamics (model-based simulations) of circuits with dual (solid line) or negative autogenous regulation (dotted line) at short times. Note the inflexion point (black point) where the dynamics changes its curvature and promoter activity is maximal (red line, normalized to help visualization; dotted red line corresponds to the negative autoregulation), while the highlighted (boost) region associates to increasing promoter activity. Parameter values from Table S1.

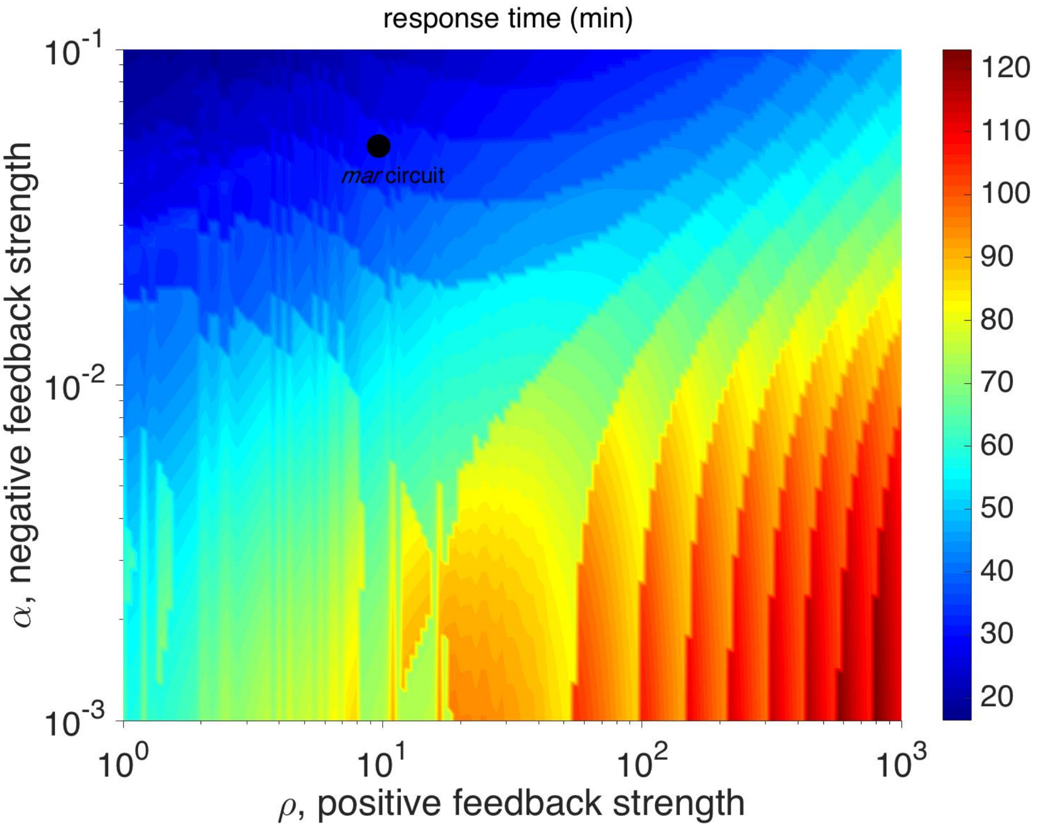

Figure S4: Analysis of the response time of the system as a function of the strength of the antagonistic feedback loops.

Heat map of the response time, calculated as the time to reach the 50% of the steady state value (t_{50}) upon induction with [*Sal*] = 5 mM. We studied the effect of ρ (proxy of the strength of the positive feedback) and α (proxy of the strength of the negative feedback). We took the parameter values from Table S1, except for π_0 because here we considered $\pi_0\,\rho = 1$ (constant) to maintain the same expression level in all cases. This plot shows, on the one hand, that when α approaches to 0 the response time increases because of the lack of active repressor. On the other hand, it shows that when ρ increases the response time also does because the dynamics is delayed.

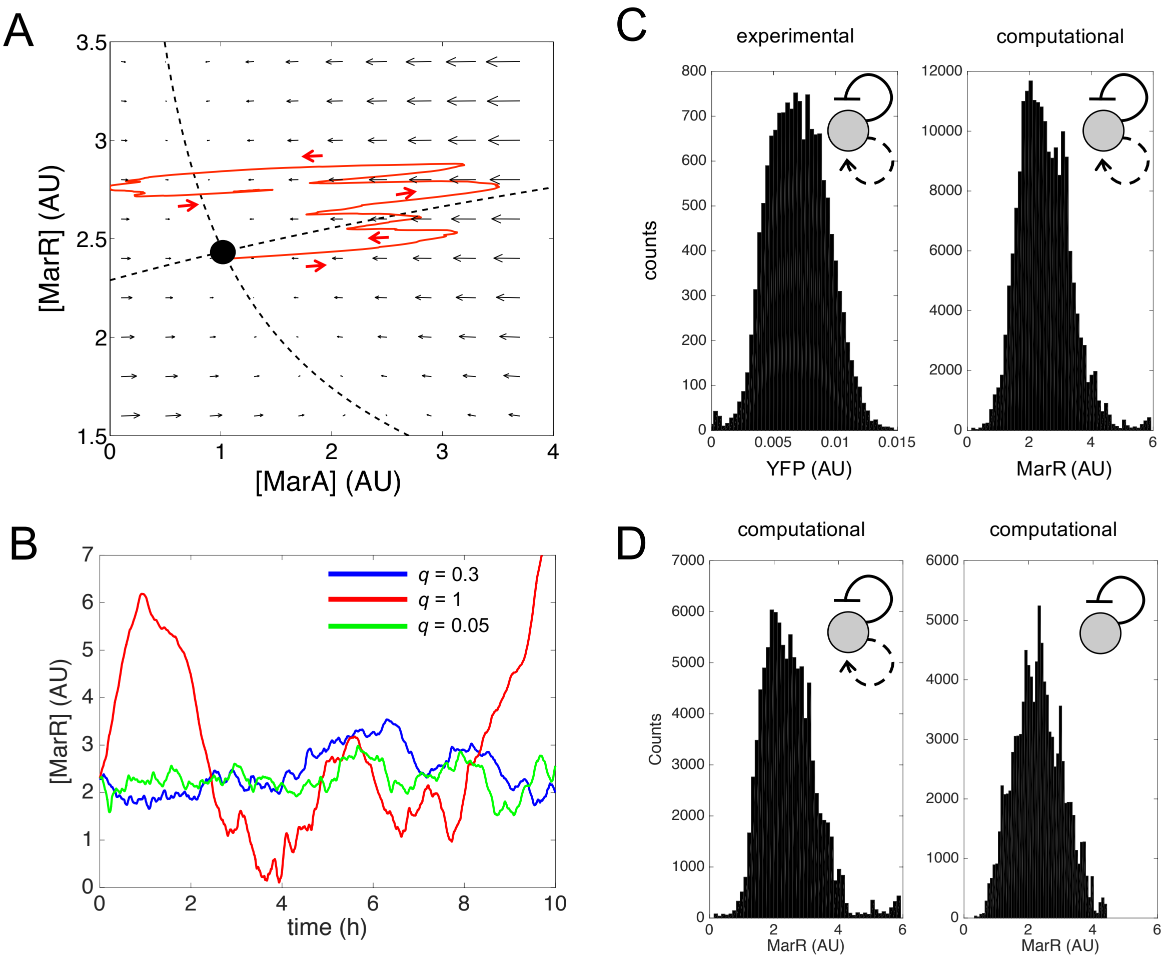

Figure S5: Non-induced stochastic dynamics (computational simulations) of the system with antagonistic autoregulation.

(A) We plot part of a trajectory in the phase space of the system (red arrows indicate time evolution). This representation highlights how gene expression levels (Δ*rob* system) fluctuate around the deterministic steady state (black point) by the combined action of intrinsic/extrinsic noise, and antagonistic autogenous control. Dashed lines correspond to the nullclines of the system, and field arrows denote the strength of change towards the steady state (bigger arrow implies bigger change). Parameter values from Table S1.

(B) Temporal simulation of the stochastic dynamics of MarR for different values of the parameter q, controlling for the amplitude of extrinsic noise. Of course, the higher the value of q, the higher the fluctuations.

(C) The distribution of YFP for all single cells at all time points confirms a unimodal, normal-like distribution (left, CV = 0.33). For this plot, we subtracted a background fluorescence of 0.055. The simulation of the stochastic dynamics of MarR also gives a unimodal, normal-like distribution (right, CV = 0.33). CV, coefficient of variation.

(D) Theoretical analysis of the non-induced dynamic response with the actual model of antagonistic autoregulation (left, CV = 0.33), and with a model without positive feedback (right, CV = 0.32; Δ*marA* scenario).

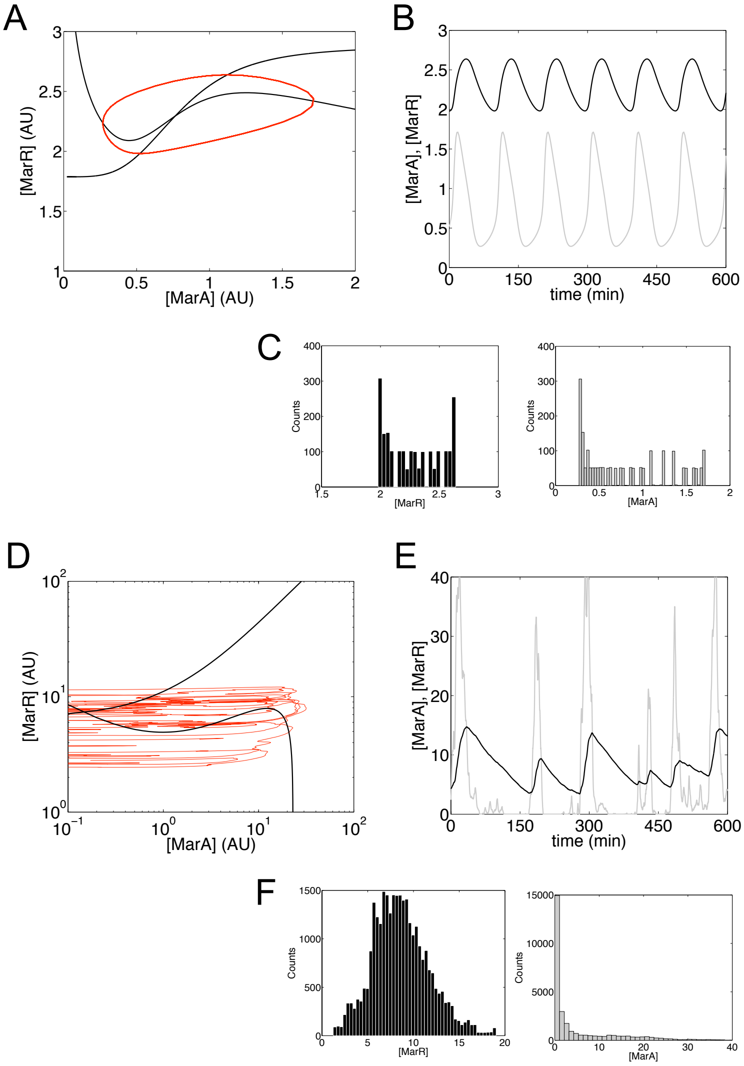

Figure S6: Theoretical analysis of the non-induced dynamic response with a nonlinear positive feedback.

(A - C) Phase diagram of the system in absence of salicylate showing an oscillatory pattern when MarA is cooperative (A). Here, we assumed high cooperativity (Hill coefficient of 4) for both MarA and MarR. The black lines correspond to the nullclines, with a different shape than for the natural system. The red curve represents a deterministic trajectory. The dynamics is represented with time in (B), where MarA is shown in gray and MarR in black. In (C) we show the resulting distributions of

expression. For these plots, we took the following parameter values: δ = 0.15 min^{-1}, μ = 0.01 min^{-1}, π_0 = 0.2 min^{-1}, ρ = 10, κ = 1, and β = 5.

(D - F) Phase diagram of the system in absence of salicylate showing stochastic pulses when MarA prevents MarR binding (D). To model this hypothetical competitive binding between MarA and MarR, we replaced y_0 in Eqs. (S4) by $y_0/(1+x)$. The black lines correspond to the nullclines, with a different shape than for the natural system. The red curve represents a stochastic trajectory. The dynamics is represented with time in (E), where MarA is shown in gray and MarR in black. In (F) we show the resulting distributions of expression. For these plots, we took the parameter values shown in Table S1. Note that whilst competition between regulators can lead to pulsatile dynamics with different periods and amplitudes, the non-competitive scenario, provided some nonlinearity, mainly shows a characteristic period and a limited amplitude (Munteanu *et al.*, 2010).

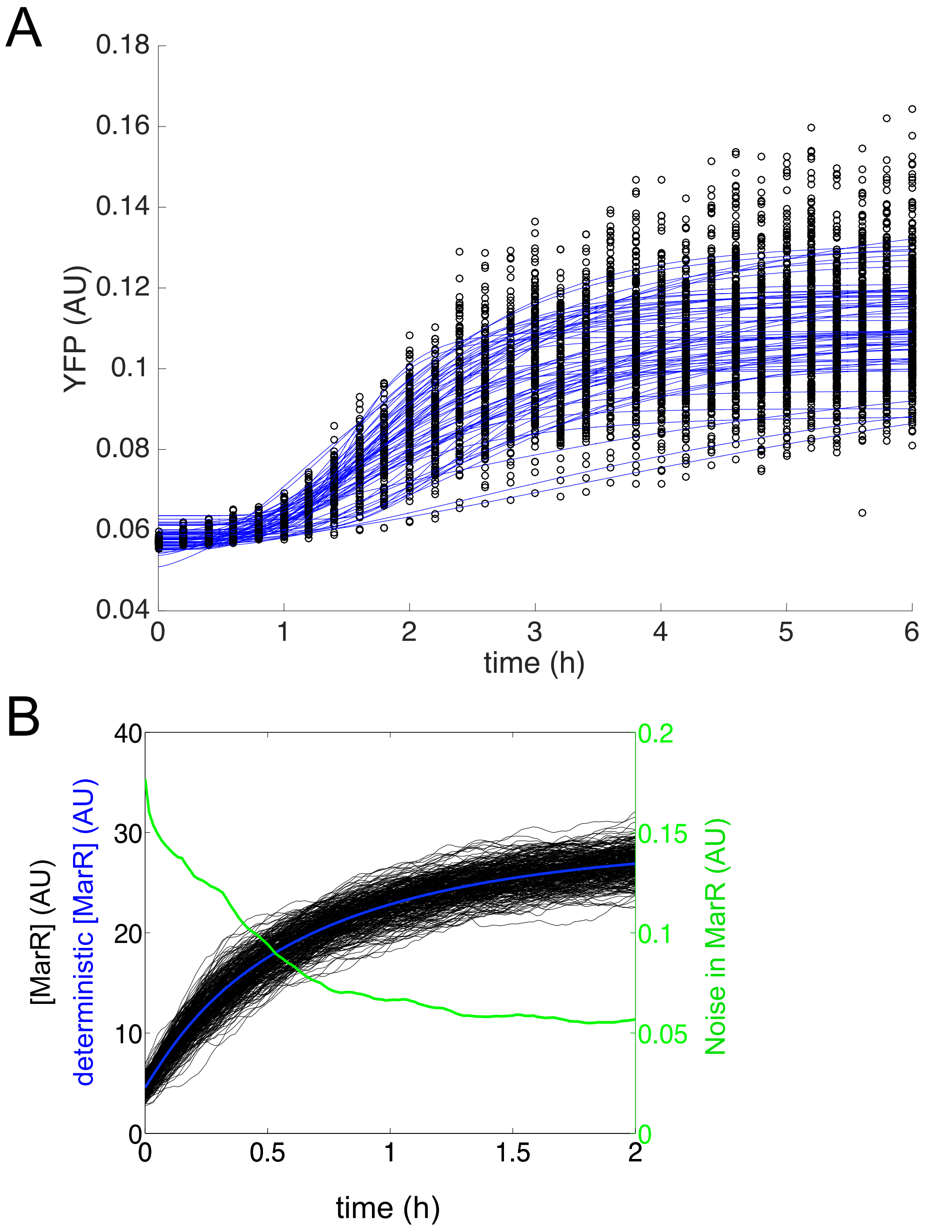

Figure S7: Individual dynamic response (antagonistic autoregulation upon induction) at the single cell level.

(A) Monitoring of fluorescence with time of all analyzed lineages (Δ*rob* strain, IE02; 5 mM salicylate) showing different response times. A lineage starts from one cell (or few) and grows and divides with time to form a colony. For each lineage we fitted (blue curves) the exponential model $\left(1-e^{-\lambda t}\right)^m$, having normalized the dynamics to go from 0 to 1. We then obtained the t_{50} values.

(B) Model simulations to study the stochastic response upon induction with salicylate. Different trajectories (simulating different single cells) are shown in black. In green, we represent noise in gene expression (coefficient of variation). In blue, we show the dynamics in the deterministic regime (which approximates very well to the average).

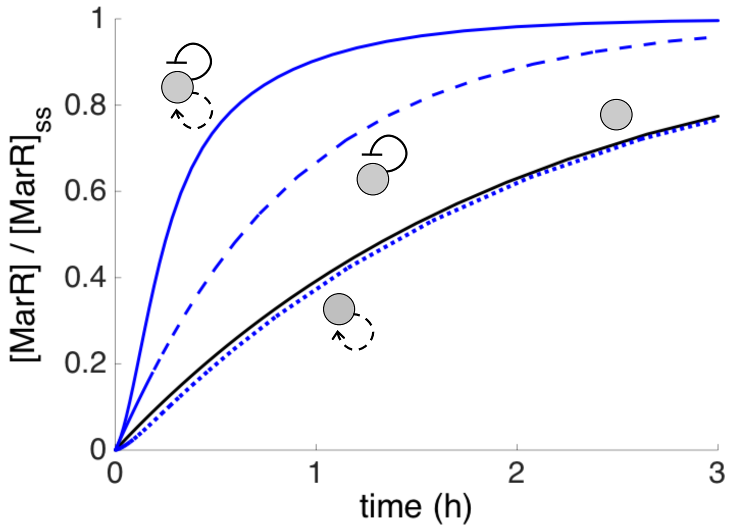

Figure S8: Theoretical analysis of the dynamics of the *mar* circuit and associated modifications when the binding of the activator and the repressor is considered competitive.

MarR response upon induction with 0.5 mM salicylate normalized by the steady state (ss) value (starting from [MarR] = [MarA] = 0 in the absence of the signal). We compared the dynamics of the wild-type circuit (solid blue line) with those of two circuit variants without the positive (dashed blue line; a circuit lacking MarA) or negative (dotted blue line; a circuit lacking MarR in presence of salicylate) feedback, respectively, and with a third system exhibiting a constitutively controlled response

(black line, this represents a null reference model of the dynamics, see main text). All simulations performed by using nominal parameter values (Table S1).

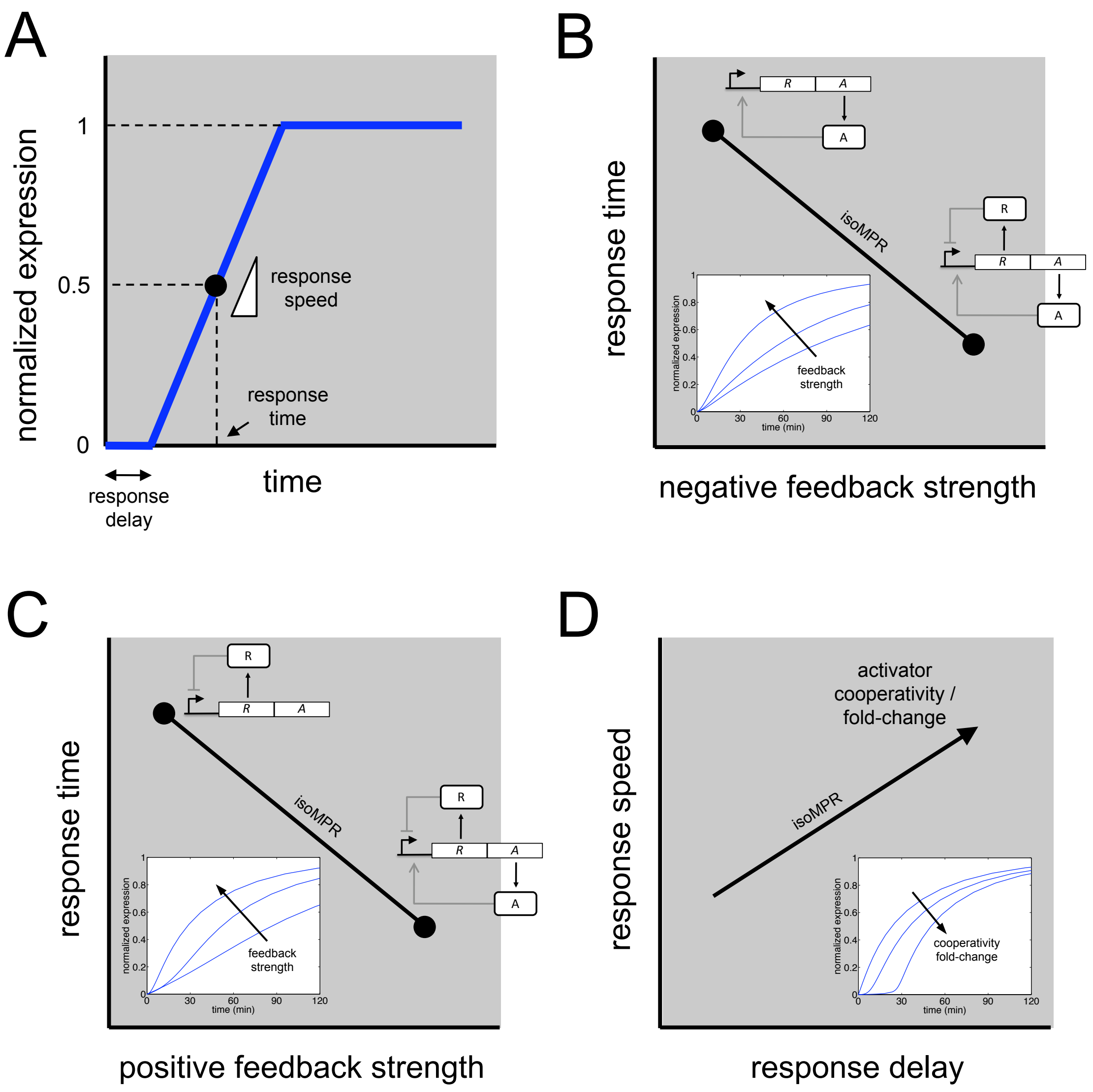

Figure S9: Analysis of the dynamic response of a circuit with antagonistic autoregulation (we used the model of the *mar* circuit).

(A) Scheme of the dynamic response.

(B) The response time depends with a decreasing trend on the negative feedback strength (understood as the binding affinity of the repressor to the promoter), being constant the maximal production rate of the operon (isoMPR). The inset shows model simulations. Note that here the repressor is a dimer.

(C) The response time depends with a decreasing trend on the positive feedback strength (understood as the binding affinity of the activator to the promoter). The inset shows model simulations. Note that here the activator is a monomer.

(D) The response speed and response delay are positively correlated and they depend with an increasing trend on the degree of multimerization of the activator (cooperativity) and its ability to stimulate of RNA polymerase (fold-change). The inset shows model simulations.

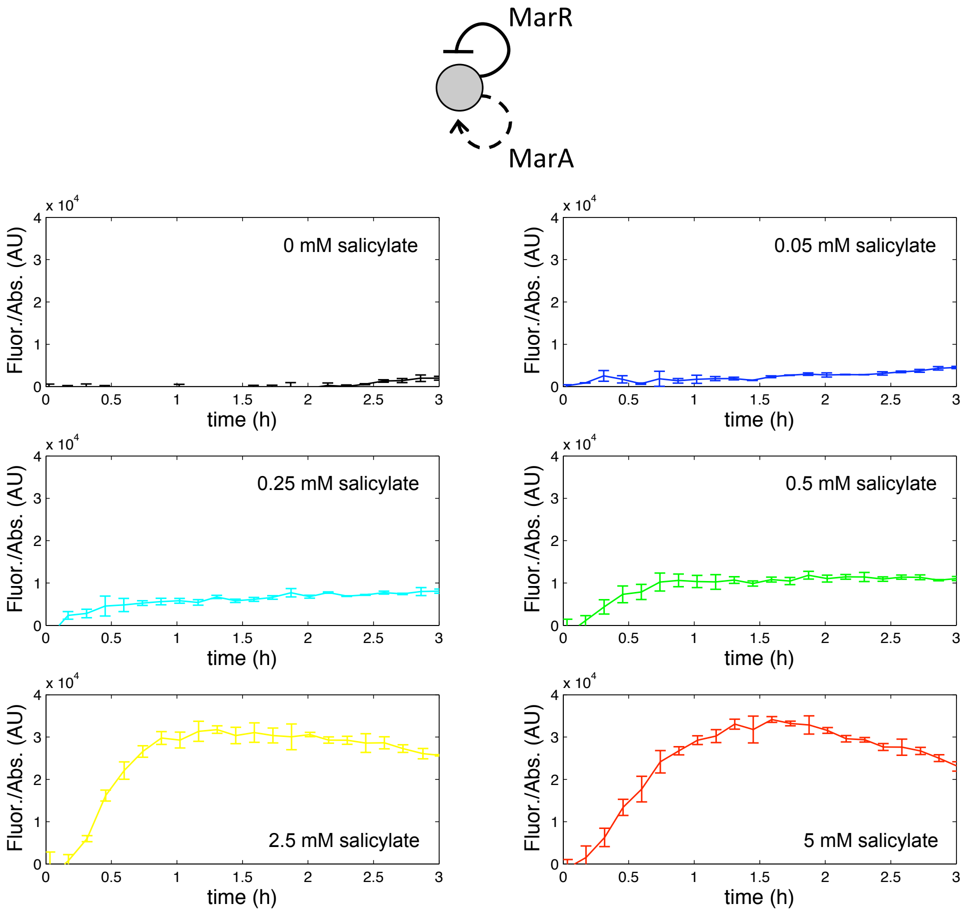
MarR
MarA
0 mM salicylate
0.05 mM salicylate
0.25 mM salicylate
0.5 mM salicylate
2.5 mM salicylate
5 mM salicylate
Fluor./Abs. (AU)
time (h)
x 10^4

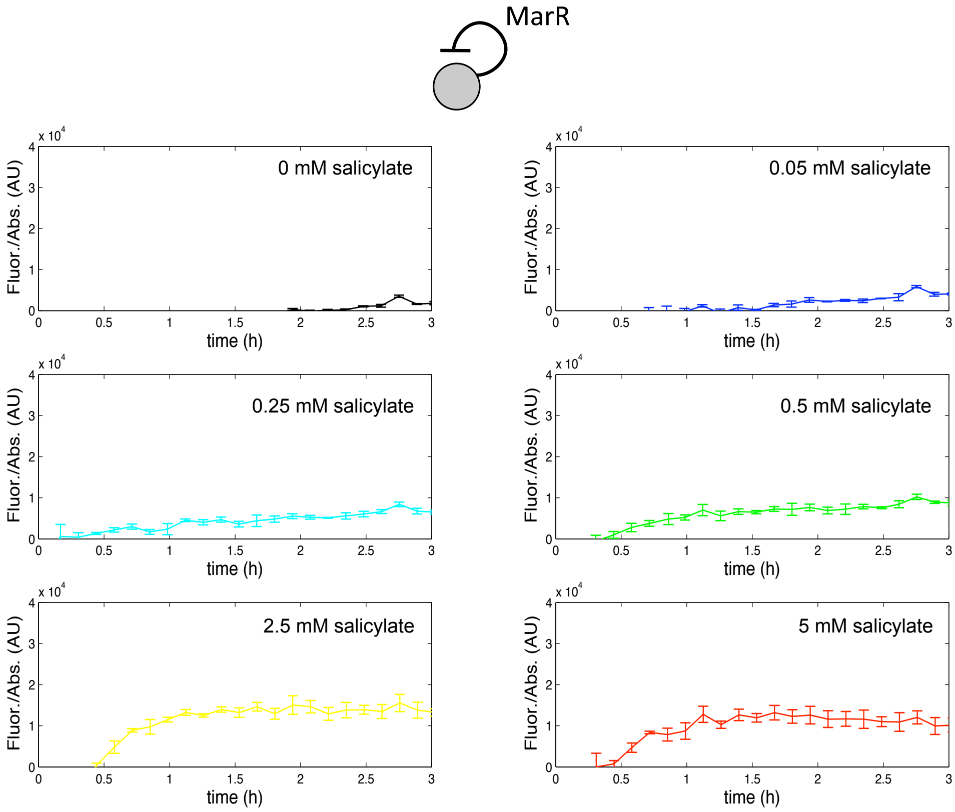
MarR
0 mM salicylate
0.05 mM salicylate
0.25 mM salicylate
0.5 mM salicylate
2.5 mM salicylate
5 mM salicylate
Fluor./Abs. (AU)
time (h)
x 10^4

Figure S10: Experimental results of the dynamic behavior with dual and single autoregulation.

Dynamic response of the system upon induction with different concentrations of salicylate (top, Δ*rob* system; bottom, Δ*rob*Δ*marA* system). We represent the normalized fluorescence (YFP) with time. Error bars represent standard deviations.

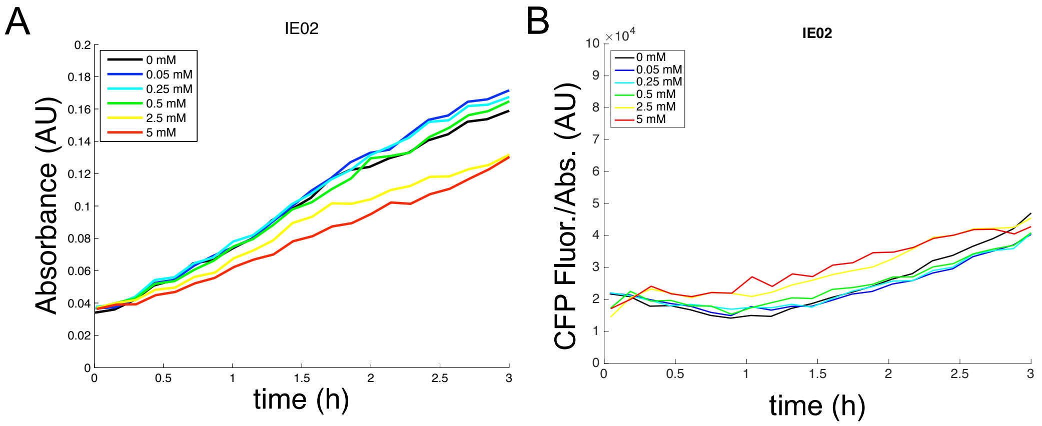

Figure S11: Experimental results of control to assess the dynamic behavior of the system.

(A) Growth curves for different levels of salicylate (Δ*rob* system). Cells are in exponential growth phase up to 3 h (then they start entering in saturation), with $R^2 >$ 0.94 in all cases by fitting Absorbance to exp(μt). For 5 mM salicylate, we obtained μ = 0.43 h^{-1} and R^2 = 0.98. These curves also show that salicylate (especially those levels higher than 1 mM) produces a cost.

(B) Normalized CFP fluorescence with time (Δ*rob* system). CFP is expressed from a constitutive promoter, then its expression should be constant with time and

independent of salicylate. These curves show that there is a slight dependence with time and salicylate, perhaps influenced by evaporation or by the growth rate (Klumpp *et al.*, 2009). However, this has not a significant impact on the dynamics of the *mar* circuit.

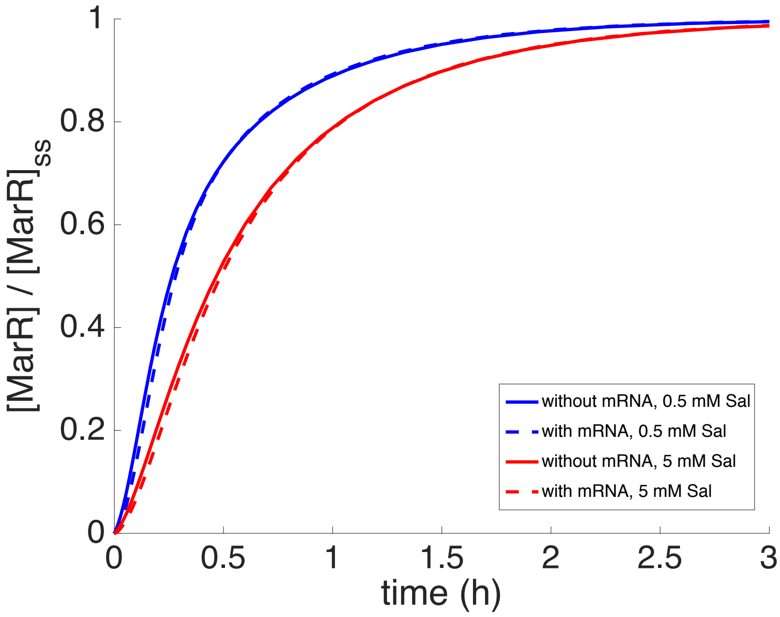

Figure S12: Theoretical analysis of the dynamics of the *mar* circuit.

Comparison of the dynamics of the *mar* circuit obtained from the extended model including mRNA [Eqs. (S6)] and from the actual model [Eqs. (S4)]. Simulations done for 0.5 and 5 mM salicylate. Parameter values from Table S1. Very similar dynamics are shown.

Supplementary Tables

Table S1: Nominal parameter values for the mathematical model.

Parameter	Value	Note	Reference
δ	0.5 min^{-1}	Protein half-life of ~1 min.	Griffith *et al.*, 2004
μ	0.007 – 0.01 min^{-1}	-	This work
π_0	0.1 min^{-1}	Assuming transcription rate of ~1 nM/min, translation rate of ~0.05-0.1 min^{-1}, and mRNA degradation rate of ~0.1 min^{-1}	Levine *et al.*, 2007
β	30	With RBS calculator	Salis *et al.*, 2009
ρ	10	Experimental data show ~6-fold increase in expression due to the direct effect of MarA.	Martin *et al.*, 1996<br>Chubiz *et al.*, 2012
κ	0.02	Having K_A = 1000-1500 nM and K_R = 5-10 nM (as $\kappa = K_R / K_A$)	Martin *et al.*, 2008<br>Seoane *et al.*, 1995
θ_S	0.13 mM	Adjusted to get the experimental transfer function. Estimated from $K_S \approx 0.9$ mM, knowing that $\theta_S \approx K_S (2\alpha)^{1/\nu_S}$	Cohen *et al.*, 1993
ν_S	1.4	-	This work
α	0.05	Assuming $[Cu^{2+}] / [MarR] \approx 5$ upon induction with 5 mM salicylate	Hao *et al.*, 2014
q	0.3	Knowing that $q = \eta_{ex}\, \pi_{mar} (2/\mu)^{1/2}$	Swain *et al.*, 2002

Supplementary References

Alekshun MN, Levy SB (1997) Regulation of chromosomally mediated multiple antibiotic resistance: the mar regulon. *Antimicrob Agents Chemother* 41: 2067-2075.

Baba T, Ara T, Hasegawa M, Takai Y, Okumura Y, Baba M, Datsenko KA, Tomita M, Wanner BL, Mori H (2006) Construction of Escherichia coli K-12 in-frame, single-gene knockout mutants: the Keio collection. *Mol Syst Biol* 2: 2006.0008.

Bedeaux D (1977) Equivalence of the master equation and the Langevin equation. *Phys Lett A* 62: 10-12.

Bintu L, Buchler NE, Garcia HG, Gerland U, Hwa T, Kondev J, Phillips R (2005) Transcriptional regulation by the numbers: models. *Curr Opin Genet Dev* 15: 116-124.

Chubiz LM, Glekas GD, Rao CV (2012) Transcriptional cross talk within the mar-sox-rob regulon in Escherichia coli is limited to the rob and marRAB operons. *J Bacteriol* 194: 4867-4875.

Cohen SP, Levy SB, Foulds J, Rosner JL (1993) Salicylate induction of antibiotic resistance in Escherichia coli: activation of the mar operon and a mar-independent pathway. *J Bacteriol* 175: 7856-7862.

Datsenko KA, Wanner BL (2000) One-step inactivation of chromosomal genes in Escherichia coli K-12 using PCR products. *Proc Natl Acad Sci USA* 97: 6640-6645.

Drepper T, *et al.* (2007) Reporter proteins for in vivo fluorescence without oxygen. *Nat Biotechnol* 25: 443-445.

Gillespie DT (2000) The chemical Langevin equation. *J Chem Phys* 113: 297-306.

Griffith KL, Shah IM, Wolf RE (2004) Proteolytic degradation of Escherichia coli transcription activators SoxS and MarA as the mechanism for reversing the induction of the superoxide (SoxRS) and multiple antibiotic resistance (Mar) regulons. *Mol Microbiol* 51: 1801-1816.

Hao Z, Lou H, Zhu R, Zhu J, Zhang D, Zhao BS, Zeng S, Chen X, Chan J, He C, Chen PR (2014) The multiple antibiotic resistance regulator MarR is a copper sensor in Escherichia coli. *Nat Chem Biol* 10: 21-28.

Klumpp S, Zhang Z, Hwa T (2009) Growth rate-dependent global effects on gene expression in bacteria. *Cell* 139: 1366-1375.

Levine E, Zhang Z, Kuhlman T, Hwa T (2007) Quantitative characteristics of gene regulation by small RNA. *PLoS Biol* 5: e229.

Martin RG, Jair KW, Wolf RE Jr, Rosner JL (1996) Autoactivation of the marRAB multiple antibiotic resistance operon by the MarA transcriptional activator in Escherichia coli. *J Bacteriol* 178: 2216-2223.

Martin RG, Rosner JL (2004) Transcriptional and translational regulation of the marRAB multiple antibiotic resistance operon in Escherichia coli. *Mol Microbiol* 53: 183-191.

Martin RG, Bartlett ES, Rosner JL, Wall ME (2008) Activation of the Escherichia coli marA/soxS/rob regulon in response to transcriptional activator concentration. *J Mol Biol* 380: 278-284.

Miyashiro T, Goulian M (2007) Stimulus-dependent differential regulation in the Escherichia coli PhoQ-PhoP system. *Proc Natl Acad Sci USA* 104: 16305-16310.

Munteanu A, Constante M, Isalan M, Solé RV (2010) Avoiding transcription factor competition at promoter level increases the chances of obtaining oscillation. *BMC Syst Biol* 4: 66.

Rensing C, Grass G (2003) Escherichia coli mechanisms of copper homeostasis in a changing environment. *FEMS Microbiol Rev* 27: 197-213.

Rodrigo G, Carrera J, and Jaramillo A (2011) Computational design of synthetic regulatory networks from a genetic library to characterize the designability of dynamical behaviors. *Nucleic Acids Res* 39: e138.

Rodrigo G, Kirov B, Shen S, and Jaramillo A (2013) Theoretical and experimental analysis of the forced LacI-AraC oscillator with a minimal gene regulatory model. *Chaos* 23: 025109.

Salis HM, Mirsky EA, Voigt CA (2009) Automated design of synthetic ribosome binding sites to control protein expression. *Nat Biotechnol* 27: 946-950.

Schneiders T, Levy SB (2006) MarA-mediated transcriptional repression of the rob promoter. *J Biol Chem* 281: 10049-10055.

Seoane AS, Levy SB (1995) Characterization of MarR, the repressor of the multiple antibiotic resistance (mar) operon in Escherichia coli. *J Bacteriol* 177: 3414-3419.

Setty Y, Mayo AE, Surette MG, Alon U (2003) Detailed map of a cis-regulatory input function. *Proc Natl Acad Sci USA* 100: 7702-7707.

Swain PS, Elowitz MB, Siggia ED (2002) Intrinsic and extrinsic contributions to stochasticity in gene expression. *Proc Natl Acad Sci USA* 99: 12795-12800.

Vinué L, McMurry LM, Levy SB (2013) The 216-bp marB gene of the marRAB operon in Escherichia coli encodes a periplasmic protein which reduces the transcription rate of marA. *FEMS Microbiol Lett* 345: 49-55.

Weiss DS, Chen JC, Ghigo JM, Boyd D, Beckwith J (1999) Localization of FtsI (PBP3) to the septal ring requires its membrane anchor, the Z ring, FtsA, FtsQ, and FtsL. *J Bacteriol* 181: 508-520.
